# Teambasierte Telemedizin im Strafvollzug – Videokonsultationen als Ansatz für Versorgungsgerechtigkeit?

**DOI:** 10.1007/s00103-025-04155-w

**Published:** 2025-11-07

**Authors:** Martin Scherer, Friedrich Völker

**Affiliations:** 1 Institut und Poliklinik für Allgemeinmedizin, Universitätsklinikum Hamburg-Eppendorf, Martinistraße 52, 20246 Hamburg, Deutschland; 2 A+ Videoclinic GmbH, Gräfelfing, Deutschland

**Keywords:** Telemedizin, Videokonsultation, Justizvollzug, Substitution, Qualitätssicherung, Telemedicine, Video consultation, Correctional care, Substitution therapy, Quality assurance

## Abstract

Videokonsultationen ermöglichen unter den strukturell erschwerten Bedingungen des Justizvollzugs eine leitlinienbasierte und kontinuierliche medizinische Versorgung. In diesem Beitrag wird das Modell der teambasierten Telemedizin anhand konkreter Einsatzfelder wie Teleallgemeinmedizin, Telepsychiatrie, Telesubstitutionstherapie und Teledermatologie vorgestellt. Besondere Beachtung finden technische Voraussetzungen, Qualitätssicherungsmaßnahmen sowie die Herausforderungen und Chancen im Versorgungsvollzug. Die dargestellten Ansätze basieren auf Erfahrungen der „Videoclinic“ in über 120 Justizvollzugsanstalten. Ergänzend werden die Ergebnisse einer ersten empirischen Analyse telemedizinischer Routinedaten (01/2023–03/2025) sowie einer Zufriedenheitsbefragung des medizinischen und pflegerischen Personals (12.03.–09.04.2024) präsentiert, die Aufschluss über die Versorgungsrealität, das Krankheitsspektrum der Inhaftierten sowie die Wirksamkeit und Akzeptanz der telemedizinischen Versorgung im Strafvollzug geben können.

## Abstract (en)

Video consultations enable guideline-based and continuous medical care under the structurally challenging conditions of correctional facilities. This article presents the model of team-based telemedicine using concrete fields of application such as tele-general medicine, telepsychiatry, tele-substitution therapy, and teledermatology. Special attention is given to technical requirements, quality assurance measures, and the challenges and opportunities in correctional healthcare delivery. The approaches described are based on the experiences of the “Videoclinic” in more than 120 correctional institutions. In addition, the results of an initial empirical analysis of telemedicine routine care data (January 2023 to March 2025) and a satisfaction survey of medical and nursing staff (March 12 to 9 April 2024) are presented, which provide insights into the realities of healthcare delivery, the disease spectrum of inmates, and indications regarding the effectiveness and acceptance of telemedicine care in correctional settings.

## Hintergrund

Inhaftierte Menschen haben gemäß dem sogenannten Äquivalenzprinzip (§§ 56 ff. StVollzG) Anspruch auf eine medizinische Versorgung, die in Umfang und Qualität der Regelversorgung außerhalb des Strafvollzugs entspricht. In der Praxis jedoch erschweren strukturelle, logistische und personelle Barrieren eine kontinuierliche, leitliniengerechte Versorgung erheblich. Viele Justizvollzugsanstalten (JVAs) verfügen nur über begrenzte ärztliche Präsenzzeiten, leiden unter Fachkräftemangel oder haben keinen Zugang zu Fachärzten und Diagnostik. Gleichzeitig ist der medizinische Bedarf hoch: Die Gefängnispopulation weist überdurchschnittlich viele chronische, psychische und suchtbedingte Erkrankungen auf.

Vor diesem Hintergrund rücken telemedizinische Verfahren in den Fokus – insbesondere die ärztliche Videokonsultation als Mittel zur Verbesserung von Erreichbarkeit und Versorgungskontinuität. In mehreren Bundesländern ist seit 2018 ein strukturiertes, teambasiertes Modell entstanden, das Videokonsultationen in JVAs regelhaft implementiert. Es liegen bereits Erfahrungen aus 120 JVAs vor. Der Artikel beschreibt das Versorgungskonzept, reflektiert seine Potenziale und Herausforderungen und enthält einen kleinen empirischen Auswertungsteil.

## Versorgungsrealität und Problemstruktur im Strafvollzug

Die gesundheitliche Situation Inhaftierter ist durch ein komplexes Belastungsprofil gekennzeichnet: Neben chronischen somatischen Erkrankungen treten psychische Störungen, Suchterkrankungen, Infektionskrankheiten und Traumatisierungen gehäuft auf [1]. Gleichzeitig bestehen systemimmanente Zugangshürden. Die ärztliche Versorgung im Vollzug ist oft an institutionelle Rahmenbedingungen gebunden, organisatorisch überlastet und strukturell isoliert.

Klassische Präsenzsprechstunden oder externe Facharztvorstellungen sind organisatorisch aufwendig, sicherheitsrelevant und vielfach unterbesetzt. Transporte erfordern Justizpersonal, führen zu Verzögerungen und erzeugen Frustration bei allen Beteiligten. Die Entkopplung von Ort, Zeit und Personalbindung, wie sie telemedizinische Verfahren bieten, erscheint unter diesen Bedingungen als logische Erweiterung des medizinischen Instrumentariums.

## Teambasierte Telemedizin im klinischen Alltag

Mit der „Videoclinic“ wurde eine Versorgungsstruktur etabliert, die Videokonsultationen systematisch in den Justizvollzug integriert. Das Modell basiert auf einer arbeitsteiligen Organisation: Pflegekräfte vor Ort begleiten die Konsultationen, dokumentieren Vitalwerte, setzen Diagnostikgeräte ein (z. B. digitale Stethoskope, Otoskope, Dermatoskope) und sichern den technischen Ablauf. Die Ärztinnen und Ärzte führen die Anamnese, Untersuchung und Therapieplanung per Video durch. Wo in einer teleallgemeinmedizinischen Konsultation die Grenze zu ziehen ist, hängt von vielfältigen situativen Gegebenheiten und letztlich der Einschätzung von Videoärztin oder -arzt ab. Nach Beauftragung durch inzwischen 6 Bundesländer liegt eine umfängliche Fallsammlung vor, aus der eine videomedizinische Realisierbarkeit von rund 94 % hervorgeht. Sechs Prozent der Fälle mussten entweder eingewiesen oder überwiesen werden (eigene Auswertung, siehe empirischer Teil dieses Artikels).

Die Sprechstunden sind werktäglich planbar und werden durch telemedizinische Bereitschaftsdienste in der Nacht, an Wochenenden und Feiertagen ergänzt.

## Telemedizinische Fachbereiche im Strafvollzug

### Teleallgemeinmedizin.

Die teleallgemeinmedizinische Versorgung umfasst Erstkontakte, Verlaufskontrollen, Medikamentenverordnungen und die Koordination von Diagnostik oder stationären Einweisungen [2]. Die Anbindung an medizinisches Fachpersonal außerhalb der JVA reduziert Wartezeiten, verbessert Behandlungsoptionen und stabilisiert die Abläufe im Pflegealltag der Anstalten.

Im Folgenden wird das *Fallbeispiel* eines Inhaftierten mit Schlafstörungen und unangemessenem Verhalten dargestellt:

Ein 19-jähriger Inhaftierter wird in der regulären Videosprechstunde vorgestellt. Die Pflegekraft berichtet, dass er sich eben erst angemeldet habe und nicht warten könne. Sie habe Rücksprache mit den Beamten seines Unterbringungsbereichs gehalten, die über anhaltende Probleme in den Abendstunden berichtet hätten. Der Inhaftierte würde sich regelmäßig zur Nacht melden, sei dann sehr unruhig, auch ängstlich, manchmal auch aggressiv, fordernd nach „Medizin“ oder einem Arzt und ließe sich nur schwer beruhigen. Tagsüber sitze er eher teilnahmslos in seiner Zelle. Deutschkenntnisse lägen keine vor, Englisch gebrochen.

Kurz nach Eintritt in das Untersuchungszimmer schildert er sein Problem so: „I can’t sleep, headache in the evening, pain in my back! I sleep only for moments, during day very tired.“ Beim Blick in die Krankenakte sind dort eine Anpassungsstörung sowie eine Depression und Angst vermerkt. Seine Medikation besteht aktuell seit seiner Inhaftierung vor 3 Wochen aus: Quetiapin 300 mg, Doxepin 50 mg abends, Lorazepam 1 mg bei Bedarf zur Nacht. Diese Medikation habe er vom aufnehmenden Arzt hier in der Justizvollzugsanstalt erhalten, der jetzt in Urlaub sei, so die Pflegekraft. Zu Beginn habe er einen eher niedergedrückten Eindruck gemacht. Besuch empfängt er nicht, Angehörige habe er nicht angegeben. Weitere Angaben sind nicht zu eruieren, Drogen nehme er keine, Alkohol verneinte er auch. Vorerkrankungen gab er nicht an. Andere Wünsche äußerte er nicht.

Telemedizinischer Befund: guter Allgemein- und Ernährungszustand, Blutdruck 120/80 mm Hg, Puls 68/min. Der äußere Eindruck ist unauffällig, Gang und Bewegungsmuster unauffällig, freie Beweglichkeit von Halswirbelsäule und Rücken. Ansonsten sind keine Auffälligkeiten zu ermitteln.

Wir bieten an einen Dolmetscher (persisch) zuzuschalten, der nach Rücksprache in 30 min zur Verfügung stehen kann. Der Patient lebt sichtlich auf, als er die Sprache hört und auf die Frage nach der Familie bricht er in Tränen aus und erzählt traumatische Erlebnisse. Sein Vater wurde getötet, von seiner Mutter und seinen beiden Geschwistern, die verschleppt wurden, hat er nach seiner Flucht nichts mehr gehört. Er hat sich alleine durchgeschlagen und kennt in Deutschland im Grunde genommen niemanden. Hier ist er in „kriminelle Kreise“ geraten.

Es folgt die Abfrage der Red Flags bei Kopf- und Rückenschmerzen und eine Exploration auf Suizidalität. Nach längerem Zuhören kommt es dann zu einem „annehmenden und aufbauenden“ Gespräch in dem Sinne, dass sich hier im medizinischen Bereich um seine Anliegen gekümmert wird. Er konnte akzeptieren, dass seine Schlaf- und Schmerzprobleme eine Folge seiner traumatischen Erlebnisse sind. Es wurde ihm angeboten, dass er sich jederzeit melden kann und er zeitnah einen Termin bei einer Fachärztin oder einem Facharzt für Psychiatrie erhält (mit Dolmetscher). Solange sollte die Medikation zunächst bestehen bleiben. Ihm wurde geraten, auf Lorazepam zu verzichten. Am Ende der Konsultation ist der Patient erleichtert und stimmt diesem Vorgehen zu.

Zufällig gab es eine Wiedervorstellung nach 4 Monaten wegen einer Sportverletzung. Es ging ihm besser, die psychiatrischen Termine fanden alle 3–4 Wochen statt. Hinsichtlich der Arzneimitteltherapie nahm er Mirtazapin 30 mg abends und bei Schmerzen 1–2 Ibuprofen 400.

In diesem Fallbeispiel konzentrierten sich die Anamnese und die körperliche Untersuchung auf neurologische und psychiatrische Aspekte. Mithilfe des Dolmetschers konnten die Art und Dauer der Beschwerden und die Red Flags (z. B. Übelkeit/Erbrechen, Sehstörung, Schwindel und andere mögliche neurologische Ausfallsymptome) abgefragt werden. Die aktive Beweglichkeit ließ sich gut untersuchen, auch durch Beobachtung beim An- und Auskleiden. Mithilfe des Dolmetschers wurden dann die weiteren Aspekte wie Gang, Einbeinstand und Koordination, Zehen- und Fersenstand untersucht. Auf der Liege konnte die Pflegkraft die grobe Kraft testen und einen Meningismus ausschließen.

### Telepsychiatrie.

Die Telepsychiatrie ist ein zentrales Anwendungsfeld der Videomedizin. Diagnostische Einschätzungen zu Depression, Psychosen, suizidalen Krisen oder Anpassungsstörungen sind per Videokonsultation möglich. Psychiatrische Expertise wird dadurch auch in Anstalten verfügbar, in denen sonst monatelange Wartezeiten herrschen. Die Videokonsultation ermöglicht es, psychiatrische Einschätzungen niedrigschwellig, regelhaft und mit klaren Prozessen in den Vollzugsalltag zu integrieren [3]. Die Erfahrungen der Telepsychiatrie in der Videoclinic zeigen modellhaft: Es kann ein besserer Zugang nicht nur zu psychiatrischen Standarddiensten ermöglicht werden, die evtl. nur schwer mit Psychiaterinnen und Psychiatern vor Ort besetzt werden können (wie es in Gefängnissen der Fall ist; [4]), sondern auch zu psychiatrischen Spezialbereichen wie der Gerontopsychiatrie, Suchtmedizin und Kinder- und Jugendpsychiatrie [5]. Auch für die Versorgung von Geflüchteten ist die Telepsychiatrie – u. a. aufgrund der unkomplizierten Hinzuziehung eines Sprach- und Kulturmittlers – eine vielversprechende Anwendungsform [6].

### Telesubstitutionstherapie.

In der bundeseinheitlichen Erhebung zur stoffgebundenen Suchtproblematik im Justizvollzug wurden Daten zur Substitutionsbehandlung in Haft erhoben. Dafür wurde u. a. die Anzahl der am Stichtag substituierten Gefangenen erfasst. Am Stichtag, dem 31.03.2021, befanden sich in den 15 Bundesländern, die bei der Datenanalyse berücksichtigt werden konnten, insgesamt 8014 Gefangene (7407 männliche und 607 weibliche) im Justizvollzug, die bei Haftantritt die Kriterien der Substanzabhängigkeit erfüllten und als Hauptsubstanz entweder Opioide oder multiple Substanzen konsumierten. Zu diesem Zeitpunkt wurden 3357 Inhaftierte (2927 männliche und 430 weibliche) substituiert. Dies entspricht einer Substitutionsquote der erfassten möglichen Patientinnen und Patienten von insgesamt 41,9 % [7].

Die Substitutionstherapie opioidabhängiger Inhaftierter stellt besondere Anforderungen. Die telemedizinische Begleitung der Substitution – inklusive Aufklärung, Dosisanpassung, Monitoring und Dokumentation – erfolgt in enger Abstimmung mit Landesärztekammern und Justizministerien. Sie ermöglicht eine durchgängige, datengestützte Therapie, auch bei Haftverlegung oder Entlassung [8].

### Teledermatologie.

Zu den häufig vorkommenden entzündlichen Hauterkrankungen in deutschen Gefängnissen zählen Ekzeme verschiedenster Genese, Psoriasis oder akneiforme Krankheitsbilder [9]. Neben synchronen Videosprechstunden kommen auch asynchrone Store-and-forward-Verfahren zum Einsatz. Dabei werden medizinische Informationen – etwa Bilder, Befunde oder anamnestische Daten – zeitversetzt übermittelt und von der behandelnden Person zu einem späteren Zeitpunkt gesichtet und beurteilt. Die Möglichkeit zur unmittelbaren Befundung reduziert Folgekontakte und beschleunigt Therapieentscheidungen.

## Technische Infrastruktur, Qualitätssicherung und Evidenzbasierung

Die technische Basis der Videokonsultationen umfasst zertifizierte Plattformen, verschlüsselte Datenübertragungen und medizinische Peripheriegeräte [10]. Die Übertragung von Bildern und Befunden erfolgt strukturiert, d. h. nach einem standardisierten Ablauf über ein datenschutzkonformes Webportal mit vordefinierten Dokumentationsfeldern und festgelegten Kommunikationswegen. Das Vollzugspersonal wird geschult und in standardisierte Abläufe eingebunden. Die Systemarchitektur erlaubt auch eine kombinierte Diagnostik (Vitaldaten, visuelle Befunde, Labor) und gewährleistet eine revisionssichere Dokumentation.

Die Qualitätssicherung erfolgt über strukturierte Prozesse: standardisierte Falldokumentation, interne Fallbesprechungen, Feedbackverfahren, Supervisionen und ein technischer Helpdesk gehören zum Modell. Insbesondere müssen Prozessbeschreibungen (SOPs, „standard operating procedures“) Klarheit schaffen, wie die Zuständigkeiten verteilt sind, wann welche Rollen aktiv involviert werden müssen und in welcher Sequenz einzelne Maßnahmen der Versorgung erfolgen [11]. Ein aktives Lob- und Beschwerdemanagement ermöglicht es den Einrichtungen, Rückmeldungen zu einzelnen Konsultationen direkt an das behandelnde Team weiterzugeben. Diese werden systematisch dokumentiert und fließen in die Weiterentwicklung der Abläufe ein. Ein externer Fachbeirat begleitet indikationsspezifische Entwicklungen, etwa in der psychiatrischen Versorgung oder der Substitution.

Um die für die Regelversorgung zugeschnittenen Leitlinien auf den Kontext der Anstaltsmedizin anzuwenden, sind kontextspezifische Anpassungen notwendig. Diese betreffen einerseits einen restriktiveren Einsatz von Medikamenten, die innerhalb der Anstalt eine hohe Attraktivität für Handel und Kriminalität besitzen, sowie andererseits Empfehlungen für Präparate der ersten Wahl.

Ein *Anwendungsbeispiel* soll dies verdeutlichen:

Es gibt eingeschränkte Evidenz, dass der Wirkstoff Pregabalin bei neuropathischen Rückenschmerzen, neuropathischen Krebsschmerzen und anderen Formen neuropathischer Schmerzen wirksam ist [12]. Im Strafvollzug ist jedoch das Abhängigkeits- und Missbrauchspotenzial von Pregabalin als äußerst kritisch zu beurteilen. Eine Gabe von Pregabalin kann daher im Vollzug nur in sehr gut begründeten Ausnahmefällen, mit sehr niedriger Dosierung und unter engmaschiger Kontrolle bezüglich Fehlgebrauch erfolgen. Im Gegensatz zur Regelversorgung ist eher Gabapentin in Erwägung zu ziehen, da es aufgrund der günstigeren Pharmakokinetik mit nichtlinearer Resorptionskinetik und der geringeren Toxizität bei oraler Überdosierung vorteilhaft ist. Bei Abhängigkeitspotenzial sollte es je nach Indikation im vollzuglichen Setting berücksichtigt werden [13].

Die evidenzbasierte Anstaltstelemedizin kann insgesamt dazu beitragen, dass:

- etablierte Behandlungsprozesse in Haft mit dem aktuellen Stand des Wissens verglichen und gegebenenfalls angepasst werden [14],
- eine Standardisierung des Zugangs zu Behandlungsverfahren sowie der Behandlungsprozesse erreicht wird, da die Telemedizin in aller Regel über verschiedene Regionen und Anstalten hinweg angewendet wird,
- Shared Decision Making, also der strukturierte Einbezug der Patientin bzw. des Patienten in Behandlungsentscheidungen und Behandlungsprozesse, auch im Anstaltskontext weitestgehend umgesetzt wird mit dem Ziel, die Betroffenen bestmöglich zur aktiven Mitarbeit bei der Erreichung von Gesundheitszielen zu motivieren [15].

## Empirische Analyse von Telemedizin in JVAs

### Methodenteil

#### Evaluationsdesign und Datengrundlage.

Bei dem vorliegenden Beitrag handelt es sich um eine deskriptive Darstellung zur Anwendung eines telemedizinischen Konsultationsmodells im Justizvollzug. Grundlage der Auswertung waren aggregierte Routinedaten der Videoclinic sowie eine einmalige schriftliche Befragung des medizinischen und pflegerischen Personals in Justizvollzugsanstalten.

#### Telemedizinischen Routinedatenerhebung.

Zur quantitativen Beschreibung der implementierten telemedizinischen Versorgung wurden Behandlungsdaten der Videoclinic aus dem Zeitraum vom 01.01.2023 bis zum 31.03.2025 ausgewertet. Insgesamt wurden 63.034 telemedizinische Konsultationen durchgeführt, wobei sowohl allgemeinmedizinische, psychiatrische, substitutionsmedizinische als auch psychotherapeutische Leistungen erfasst wurden. Die erhobenen Daten ermöglichen einen Überblick über die Versorgungsrealität, das Krankheitsspektrum der Inhaftierten sowie die Akzeptanz der telemedizinischen Ansätze bei den Mitarbeitenden im Strafvollzug.

Die Behandlungsdaten wurden im Rahmen der Behandlungsdokumentation durch die telemedizinisch tätigen Ärztinnen und Ärzte in einer Software der Videoclinic erfasst. Durch die Festlegung von Pflichtfeldern und Eingabeparametern wurde sichergestellt, dass die wichtigsten Daten verlässlich bei jeder Behandlung erfasst werden. Eine Validitätskontrolle der Daten fand allerdings nicht statt. Auch konnten keine Daten der präsenzmedizinischen Versorgung durch intramural tätige Ärztinnen und Ärzte in die Analyse einbezogen werden, da kein Zugriff auf die Krankenakten der Justizvollzugsanstalten besteht.

#### Fragebogenerhebung.

Zur ergänzenden Bewertung des telemedizinischen Angebots wurde ein standardisierter Fragebogen entwickelt, der in Abstimmung mit den Justizministerien der Bundesländer an 116 Justizvollzugsanstalten übermittelt wurde. Dort erfolgte die Weiterleitung gezielt an Mitarbeitende, die mit der Videoclinic zusammenarbeiteten. Auf die Verteilung der Fragebögen in den JVAs hatten wir keinen Einfluss, sodass es sich hier um ein *Convenience Sample* handelte. Eine Filterfrage zu Beginn des Fragebogens stellte sicher, dass nur Personen mit direkter Erfahrung in die Auswertung einbezogen wurden. Die Befragung fand im Zeitraum 12.03. bis 09.04.2024 statt.

Der Fragebogen enthielt geschlossene und offene Fragen zu soziodemografischen Merkmalen, Nutzungserfahrungen, wahrgenommener Entlastung im Arbeitsalltag, Einschätzungen zur Patientensicherheit sowie zur technischen und organisatorischen Umsetzung der telemedizinischen Konsultationen. Weitere Items betrafen die Verständlichkeit der ärztlichen Dokumentation und die Zufriedenheit mit dem Schulungsangebot. Ergänzend wurden Freitextfelder für individuelle Rückmeldungen bereitgestellt.

Die Auswertung der Rückmeldungen erfolgte deskriptiv mit IBM SPSS Statistics, Version 29.0.2.0 (IBM, Armonk, NY, USA).

#### Datenschutz und ethische Aspekte.

Die Erhebung erfolgte anonym. Eine Zuordnung der Angaben zu einzelnen Personen oder Justizvollzugsanstalten war zu keinem Zeitpunkt möglich. Eine formale Abstimmung mit den Justizministerien der beteiligten Bundesländer wurde im Vorfeld eingeholt.

### Ergebnisteil

#### Telemedizinische Routinedatenerhebung.

Die wichtigsten Basisdaten zu telemedizinischen Behandlungen und zur Verteilung der Konsultationen sind in Tab. 1 zusammengefasst. Von den insgesamt 63.034 dokumentierten telemedizinischen Behandlungen entfiel der größte Anteil auf allgemeinmedizinische Konsultationen (55 %), gefolgt von psychiatrischen Konsultationen (28 %), Substitutionstherapie (10 %) und psychotherapeutischen Angeboten (5 %). Die Patientenkohorte bestand überwiegend aus männlichen Inhaftierten (89,4 %).

**Tab. 1:** Telemedizinische Routinedaten der Videoclinic (*N* = 63.034 Behandlungen im Zeitraum 01/2023–03/2025)

Variable	Ausprägung	Anzahl	Anteil (%)
Geschlecht der behandelten Person	Männlich	57.000	89,4
Fachgebiet der Behandlung	Allgemeinmedizin	34.857	55
	Psychiatrie	17.959	28
	Substitution	6044	10
	Psychotherapie	3311	5
	Andere Fachgebiete	863	1
Häufigste Diagnosen (ICD-10)	Psychische und Verhaltensstörungen durch multiple Substanzen (F19)	2076	8
	Psychische und Verhaltensstörungen durch Alkohol (F10)	1478	6
	Psychische und Verhaltensstörungen durch Opioide (F11)	1435	6
	Depressive Episoden (F32)	1354	5
	Nichtorganische Schlafstörungen (F51)	1330	5
	Reaktionen auf schwere Belastungen und Anpassungsstörungen (F43)	1227	5
	Schizophrenie (F20)	1107	4
	Psychostimulanzienbezogene Störungen (F15)	1089	4
	Cannabinoidbezogene Störungen (F12)	1086	4
	Rezidivierende depressive Störungen (F33)	799	3
Behandlungsabschluss	Telemedizinisch abschließend behandelt	59.911	94
	Überweisungen/Einweisungen notwendig	3824	6
Durchschnittliche Reaktionszeit (Bereitschaftsdienst)	–	–	<3 min

Die häufigsten Diagnosen umfassten psychische und Verhaltensstörungen durch multiple Substanzen (F19, 8 %), Alkohol (F10, 6 %) und Opioide (F11, 6 %) sowie depressive Episoden (F32, 5 %), nichtorganische Schlafstörungen (F51, 5 %) und Anpassungsstörungen (F43, 5 %). Diese Verteilung spiegelt die besondere psychosoziale Belastung der Inhaftierten wider.

Es konnten 94 % der Patientenkontakte telemedizinisch erfolgreich abgeschlossen werden, sodass nur in 6 % der Fälle eine weiterführende Überweisung notwendig war. Die durchschnittliche Reaktionszeit im Bereitschaftsdienst lag unter 3 min.

In der oben beschriebenen quantitativen Umfrage des Justizpersonals äußerten sich 84 % der Befragten zufrieden oder sehr zufrieden mit der telemedizinischen Versorgung. Zudem wurde von 80 % der Befragten eine Entlastung im Arbeitsalltag wahrgenommen.

#### Fragebogenerhebung.

Im Rahmen der standardisierten Erhebung konnten 55 vollständig ausgefüllte Fragebögen von Mitarbeitenden aus Justizvollzugsanstalten ausgewertet werden. Die befragten Personen setzten sich überwiegend aus Krankenpflegekräften und Sanitätsfachkräften zusammen; kleinere Gruppen entfielen auf ärztliches Personal sowie sonstige Berufsgruppen (Tab. 2). Die Mehrheit der Teilnehmenden war zwischen 25 und 45 Jahren alt. Hinsichtlich des Geschlechts lag ein höherer Anteil weiblicher Befragter vor.

**Tab. 2:** Charakteristika der befragten JVA-Bediensteten (*N* = 55)

Merkmal	Ausprägung	Anzahl
Alter	25–45 Jahre	34
	>45 Jahre	21
Geschlecht	Männlich	17
	Weiblich	35
	Divers	2
	Keine Angabe (Geschlecht)	1
Berufsgruppe	Krankenpflegekräfte/Sanitätsfachkräfte	41
	Ärztinnen/Ärzte	7
	Sonstige	3
	Keine Angabe	4

Die Mehrheit der Teilnehmenden war mit dem telemedizinischen Angebot der Videoclinic zufrieden oder sehr zufrieden (Tab. 3). In den Freitextkommentaren wurden die strukturierten Schulungen des Anstaltspersonals, die gute Erreichbarkeit der Hotline, die Bild- und Tonqualität der Konsultationen sowie die Justizvollzugserfahrung der telemedizinisch tätigen Ärztinnen und Ärzte positiv hervorgehoben. Auch die Reaktionsgeschwindigkeit des ärztlichen Bereitschaftsdienstes wurde mehrfach als vorteilhaft beschrieben. Kritische Rückmeldungen bezogen sich vor allem auf technische und organisatorische Schnittstellenprobleme: So wurde etwa die Bedienung der in einigen Anstalten eingesetzten Remote-Management-Devices (RMDs) als umständlich beschrieben. Außerdem wurde der manuelle Aufwand für die Übertragung der ärztlichen Dokumentation in die Anstaltsakten angesprochen, da derzeit keine direkte Schnittstelle besteht.

**Tab. 3:** Zufriedenheit mit dem telemedizinischen Angebot. Ergebnisse der Befragung von JVA-Bediensteten (*N* = 55)

Kategorie	Anzahl	Anteil (%)
Zufriedenheit Bediensteter (zufrieden oder sehr zufrieden)	46	84
Bedienstete, die die Qualität der Behandlung als vergleichbar oder besser einschätzen	36	65
Bedienstete, die Telemedizin als entlastend empfinden	44	80
Zusammenspiel zwischen Teleärztin/-arzt und JVA-Personal funktioniert gut	46	84
Gefangene sind mit dem telemedizinischen Angebot zufrieden	40	73
Patientensicherheit bei teleärztlicher Behandlung wird als hoch eingeschätzt	44	80

Die Ergebnisse zeigen zudem, dass das Zusammenspiel zwischen JVA-Personal und Teleärztin/-arzt aus Sicht der Befragten überwiegend gut funktioniert (Tab. 3). Die Mehrheit (80 %) schätzte die Patientensicherheit im Rahmen der telemedizinischen Behandlung als hoch ein. Die Qualität der telemedizinischen Behandlung wurde von 65 % als vergleichbar mit der regulären Behandlung oder als besser eingeschätzt. Auch die Zufriedenheit der Inhaftierten mit dem Angebot wurde mehrheitlich (73 %) als gegeben eingeschätzt.

### Limitationen und Ausblick

Ein wesentlicher Limitationspunkt der vorliegenden Analyse ist, dass es sich bei den präsentierten Daten um eine interne Evaluation handelt, die innerhalb der Videoclinic durchgeführt wurde. Die Befragung des JVA-Personals war unsystematisch und beruhte auf einem *Convenience Sample*. Zwar liefern diese Daten wertvolle Einblicke in die Akzeptanz und Wirksamkeit der telemedizinischen Versorgung im Justizvollzug, doch fehlen bislang unabhängige, externe Evaluationen, die eine belastbare Aussage über Wirksamkeit und Übertragbarkeit ermöglichen.

Um die Potenziale telemedizinischer Anwendungen im Justizvollzug realistisch zu bewerten, sind daher langfristige, unabhängige Studien notwendig, die sowohl Chancen als auch Risiken systematisch erfassen. Perspektivisch wäre es wünschenswert, Forschungs- und Versorgungsstrukturen zu fördern, die evidenzbasierte, praxisnahe und ethisch reflektierte Implementierungsstrategien ermöglichen.

## Chancen, Grenzen und Zukunftsperspektiven

Videokonsultationen sind kein Ersatz für die Präsenzmedizin, aber eine wertvolle Erweiterung unter strukturell erschwerten Bedingungen. Sie ermöglichen Versorgung, wo sie sonst ausfiele, und bieten eine klare Dokumentation, Planbarkeit und fachliche Rückkopplung. Auch aus justizieller Sicht – mit Blick auf Sicherheit, Personalbindung und Haftalltag – ist die telemedizinische Versorgung eine entlastende Struktur.

Grenzen bestehen bei komplexen körperlichen Untersuchungen, sprachlichen Barrieren oder akuten Notfällen. Auch bedarf es technischer Mindeststandards, funktionierender Infrastruktur und einer Einbindung des Vollzugspersonals.

Telemedizinische Konsultationen können nicht alle Herausforderungen struktureller Unterversorgung kompensieren. Gerade in Einrichtungen mit chronisch unzureichender personeller oder infrastruktureller Ausstattung kann Telemedizin bestehende Lücken bestenfalls punktuell überbrücken – nicht aber ersetzen. Ein Überstrapazieren digitaler Lösungen birgt zudem das Risiko, dass zentrale Elemente ärztlicher Versorgung – wie die direkte körperliche Untersuchung, der Beziehungsaufbau oder das Vertrauen – in den Hintergrund treten.

Das Modell ist anschlussfähig: Auch in Maßregelvollzug, Sicherungsverwahrung oder dezentralen Versorgungseinheiten kann es Anwendung finden. International zeigen Beispiele aus Finnland [16] und den USA [17] vergleichbare Entwicklungen.

## Fazit

Die teambasierte Videokonsultation kann eine tragfähige Versorgungsform im Justizvollzug darstellen. Sie schafft medizinische Nähe trotz räumlicher Distanz, integriert verschiedene Berufsgruppen, entlastet das System und sichert die Teilhabe Inhaftierter an einer menschenwürdigen Gesundheitsversorgung. Die Digitalisierung eröffnet hierbei nicht nur Effizienzgewinne, sondern fördert auch echte Versorgungsgerechtigkeit – selbst „im Knast“.
